# Malnutrition as Key Predictor of Physical Frailty among Malaysian Older Adults

**DOI:** 10.3390/nu12061713

**Published:** 2020-06-08

**Authors:** Camilla Wahida Norazman, Siti Nur’Asyura Adznam, Rosita Jamaluddin

**Affiliations:** 1 Department of Nutrition and Dietetics, Faculty of Medicine and Health Sciences, Universiti Putra Malaysia, Serdang 43400, Selangor, Malaysia; camillanorazman@yahoo.com (C.W.N.); rositaj@upm.edu.my (R.J.); 2 Malaysian Research Institute of Ageing, (MyAgeing) Universiti Putra Malaysia, Serdang 43400, Selangor, Malaysia

**Keywords:** frailty, ageing, malnutrition, elderly, sarcopenia

## Abstract

Studies have been carried out on the association between frailty and malnutrition, but the similarities and divergence of the relationship remain debatable. This study aimed to explore the prevalence of malnutrition risk and frailty as well as the overlapping constructs. The associations that emerged were assessed independently of other risk factors. A total of 301 community-dwelling older adults with a mean age of 66.91 ± 5.59 years old were randomly recruited. Fried Criteria and Mini Nutritional Assessment-Short Form (MNA-SF) were used to assess frailty status and malnutrition, respectively. Other related nutritional assessments were assessed (body mass index (BMI), circumference measures, body fat % and skeletal muscle mass). The prevalence of frailty was 14.6% and prefrail was 59.7%; 29.6% were at risk of malnutrition, and 3.3% were malnourished. Malnutrition risk was significantly associated with a higher number of chronic diseases, BMI, circumference of mid-upper arm (MUAC), and calf, (CC)and skeletal muscle mass (SMM) and frailty, whereas frailty was significantly associated with higher number of chronic diseases, SMM and malnutrition. Frailty syndrome can be predicted with increasing age, body fat, lower skeletal muscle and malnutrition. Those who were frail were found to be five times more likely to be at risk of malnutrition. Results suggested that frailty and malnutrition shared considerable overlap, which emphasised the interrelated but discrete concepts. Therefore, the assessment of malnutrition is imperative and could be used as a practical implication in assessing frailty syndrome.

## 1. Introduction

Frailty is a multidimensional syndrome that has been a crucial aspect of research within the geriatric field. It is generally considered synonymous with disability, comorbidity and other syndromes under the giant umbrella of the geriatric syndrome. From a clinical interpretation, frailty state is critical in instigating severe complications which can lead individuals to a higher risk of adverse health outcomes, such as falls, regress in mobility and independence, hospitalisation, disability and death [1]. The common characteristics of frailty are noticeable weight loss and deteriorating physical functions, exhibited from the ageing perspectives of malnutrition and anorexia.

The relationship between malnutrition and frailty has been widely discussed in the literature [2,3], which is present synergistically among older adults. Both conditions are closely associated with prognosis implications of poor health which would increase dependency, and even death, as previously implied [3,4]. Malnutrition is defined as “a state resulting from lack of uptake or intake of nutrition that leads to altered body composition (decreased fat-free mass) and body cell mass, leading to diminished physical and mental function as well as an impaired clinical outcome” [5]. Malnutrition can also be related to undernutrition [6], which is, primarily, ubiquitous to ageing in the elderly or anorexia of ageing due to a decrease in nutritional intake that results in a deficiency of muscle mass and strength. Hence, malnutrition is believed to be essential in the onset of frailty, consequently causing physical functions to deteriorate. 

Current evidence hypothesised that frailty and malnutrition are two concepts that share common determinants and several pathophysiological pathways. Some of these included reduced body tissue, presence of chronic inflammation, pernicious sociodemographic background, impaired physical and cognitive functions as well as dependence in bodily functions [3,7]. Hence, older individuals commonly show both physical frailty and malnutrition. Both Van Kan and Vellas (2011) argued that older individuals at risk of malnutrition should be regarded as frail. Nonetheless, low nutrient intake and clinical appearance of significant weight loss would exacerbate the nutrition reserved in the body against stressors [8]. 

The relationship between frailty and malnutrition were widely explored using a well-validated instrument, the mini nutritional assessment (MNA), regardless of the simple adaptation, or the original version. The assessment was validated and well accepted in screening malnutrition risks among older adults. In general, two out of three malnourished older adults were physically frail, while only about one in 10 of the population that was physically frail was malnourished [9]. A systematic review and meta-analysis using MNA revealed the prevalence of malnutrition was at 2.3%, and the prevalence of physical frailty was reported to be much higher at 19.1% [9]. In another study, half of the frail individuals were identified to be at risk of malnutrition, with more than 90% at risk of either being frail or prefrail [4]. These findings revealed that some common elements of measurement in MNA and the Fried criteria were cohesive, such as weight loss, low body mass index (BMI), and impaired physical and mental functions, which mainly explained the strong relationship of both constructs. Although closely related, malnutrition and frailty possessed unique entities that could be distinguished in terms of clinical features. 

Loss of skeletal muscle mass and skeletal muscle wasting would be evident in cases of malnutrition and frailty when the muscle circumference in the arms and calves is noticeably reduced. Previous studies suggested that reduced muscle mass was a definitive feature in loss of manual pressure strength [10,11,12]. Nonetheless, epidemiological studies conducted among the older population had reported a significant decline in anthropometric parameters, which reflected the overall health status. As a person aged, the skeletal muscle would endure a continuous loss of quality and quantity of muscle mass and strength [11]. This age-related loss of muscle mass was strongly correlated with a relative increase in fat mass [13], which could lead to sarcopenic obesity [14], which can be an essential risk factor to becoming physically frail and disabled [15,16]. As such, physical training and rehabilitation activities were proposed to be strategies that helped minimise functional impairment and improve physical strength [13,17].

### Aim of the Study

The purpose of this study was to compare the differences in demography, body composition, nutritional status and malnutrition among community-dwelling older adults residing in the capital city of Kuala Lumpur. Prevalence of malnutrition risk and frailty and overlapping constructs were explored to assess the association of other risk factors independently.

## 2. Materials and Methods 

### 2.1. Study Design

This cross-sectional study was conducted on older adults aged 60 years and above who were living in 10 randomly selected People’s Housing Projects (PPR) flats at Kuala Lumpur, the capital city of Malaysia. A cluster sampling method was applied, whereby all blocks from each chosen flats were included in the study. Census data on the residents in each apartment complex were obtained from the City Hall of Kuala Lumpur (DBKL), and the number of subjects recruited was based on proportionate sampling. Subjects were screened for inclusion and exclusion criteria selection before participation. The inclusion criteria included older adults aged 60 and above who resided in flats and were able to ambulate. Subjects were excluded if they showed a severe sensory deficit which hindered them from performing a physical functioning assessment, had an unstable medical condition or were in palliative care. Three-hundred-and-ten (310) older adults who were eligible for this study were recruited, but nine (9) were excluded due to missing data. Approval and permission in conducting the study were granted by the Ethical Research Committee, which involved the Human Research Department at Universiti Putra Malaysia and Community Development and Urban Wellbeing Department of Kuala Lumpur City Hall (DBKL). The consent form was then disseminated to the eligible subjects and signed for confirmation of their participation. The interview-based survey was conducted by trained researchers at the community centre of each apartment complex. The subjects who agreed to participate were invited to the interview sessions and reminded a day before the allocated sessions to be present. Upon inviting respondents to participate in the study, information sheet and consent forms were distributed. Further details on personal information and contact number were obtained from respondents who agreed to participate. Two days prior to data collection, respondents were reminded through phone calls to fast overnight for a minimum of eight to 12 hours. In the event whereby respondents failed to fulfil the requirement to fast overnight, a subsequent visit attempt was made where they were reminded to fast overnight a day before. 

### 2.2. Measurement

Sociodemographic data included age, gender, race, education, marital status, living arrangement, working status and monthly household income. The self-report of chronic diseases diagnosed and treated by a physician was recorded for ten (10) identified diagnoses and other disorders.

#### 2.2.1. Frailty Syndrome

Frailty syndrome was indicated based on the well-established, standardised phenotype of frailty, proposed by Fried et al. (2001). The phenotypes evaluated five components of frailty syndrome, which denoted one point for each criterion met. The first phenotype on (1) shrinking was based on the subjective report of unintentional weight loss of more than 5 kg for the past six to 12 months. The second phenotype focused on (2) exhaustion, which was assessed based on two questions of self-reporting on fatigue from the CES-D scale of depression that was proposed in the original method. (3) Weakness was identified if the handgrip strength was lower than the cut-off point proposed by Asian Working Group of Sarcopenia (AWGS). Handgrip strength was measured using the Jamar Hydraulic Hand Dyanmometer. In a sitting position, respondents were asked to hold the dynamometer vertically using their dominant hand. They were required to compress the handgrip to their maximum strength for three attempts. (4) Slowness was measured by the gait speed of walking four meters at the usual speed and those who took less than the cut-off point at 0.8 m/s is considered slow. Finally, (5) low physical activity was identified by the low score of the lowest tertile in the physical activity scale for the elderly (PASE). The Malay version of PASE was used instead of the components mentioned in the original Fried Criteria [18], which was validated by older Malaysian adults. The use of the Malay PASE questionnaire was more applicable than the Minnesota leisure time physical activity questionnaire (MLAQ) as it has been validated among the Malaysian elderly [18]. The MLAQ was originally designed for a young population, sex-specific generalised to men, and has been validated only for healthy older population [19]. The most important aspect to be highlighted is that the activities listed in the MLAQ, such as mountain climbing, water activities, winter activities, golf and hunting animals, are less relevant to the Malaysian practice and culture. Subjects who met zero criteria were defined as nonfrail, while those who met one or two criteria were defined as prefrail. Those who met more than three criteria were considered as being frail. For the present study, the modification was made on the cut-off point and the assessment of physical activity to fit the population better.

#### 2.2.2. Body Composition Assessment

Anthropometric measurements that were included in this study were weight, height and body mass index (BMI). The circumferences of the crucial body parts were also considered in the study, including waist circumference (WC), mid-upper arm circumference (MUAC), and calf circumference (CC). The invasive method using bioelectrical impedance analysis (BIA; Omron Healthcare Co., HBF-375 prototype) was used to assess the body composition parameters, namely body fat percentage, visceral fat and skeletal muscle mass. All respondents fasted for at least eight to 12 hours prior to the administration as the assessment of body composition using BIA is highly dependent on hydration status. Respondents were required to void the bladder and to avoid any vigorous activity beforehand. Participants stood bare-footed on the footplate electrodes on the analyser holding the handgrip electrodes with both hands. This device applies single-frequency (50 kHz, 500 µA) with eight sensors on both hands and feet for complete body measurement. The machine is calibrated daily and the measurements were taken twice in accordance with the standard procedure. 

#### 2.2.3. Malnutrition Assessment

The mini nutritional assessment (MNA) was employed to identify older adults who were at risk of malnutrition. Both MNA and MNA-SF (MNA-short form) are validated and effective tools for malnutrition screening [20]. Other assessment tools for malnutrition, such as GLIM and ESPEN, were mostly validated against the hospitalised population, and thus the application in the community setting should be justified with further validation [21]. This is especially true as the prevalence of secondary malnutrition is much lower in the community than the hospitalised patients [21]. Although these tools have been applied in the community setting in several studies [22,23], the criteria are still new and the lack of understanding of the association between frailty and GLIM criteria in community-dwelling elderly warrant further research in this crucial respect. 

Since the original version of MNA that was used was found to be lengthy, which would be impractical for the implementation of this study and less effective in a community setting, a shorter version was used. This shorter version was also always preferred among scholars [24]. Particularly, the MNA-SF has been found to be an effective and valid screening scale to define malnutrition in frail older adults which support the objective of this study [24]. MNA-SF have been validated against anthropometric and functional assessments among the Malaysian population, which showed high sensitivity (93.2%), specificity (79.4%) and positive predictive value (PPV) (18.2%) in detecting malnutrition risk among the Malaysian elderly [25]. MNA-SF consisted of six questions with an overall score ranging from 0–14. The range for well-nourished was between 12 and 14, while the range for at risk of malnutrition was between 8 and 11. Malnourished was at a score of 7 or less. 

### 2.3. Statistical Analysis

Data were analysed using Statistical Package for Statistical Package for Social Science (SPSS) (Version 22) All variables were assessed for homogeneity of variance before any statistical analysis was attempted. Exploratory data analysis (EDA) was used to check for data normality of all variables. In the descriptive analysis, continuous variables were expressed in mean and standard deviation (SD), while categorical variables were presented as frequency (*n*) and percentage (%) in the cross-tabulation. Subsequently, the chi-square test of association and the Pearson product moment correlation was used to compare the difference of covariates between subgroups of frailty status (robust, prefrail and frail) and nutritional status (well-nourished and at risk of malnutrition). In cases where the variables were not normally distributed, a nonparametric test of Spearman correlation was used instead. Additionally, the chi-squared test was used to observe the frequency differences between the categorical covariates with the observed variable. To determine the correlation between various risk factors with the risk of frailty syndrome, variables which were found to be significant in the bivariate analysis were further included in the regression analysis. The multivariate logistic analysis was used to determine the significant predictors for frailty syndrome. Data with a *p*-value less than or equal to 0.05 were considered to be statistically significant.

## 3. Results

### 3.1. Baseline Characteristic of the Subjects and Frailty Status

The mean age of the subjects in this study was 67.08 ± 5.536 years, ranging from 60 to 84 years old. By ethnicity, the majority of the subjects were Malay, followed by Indian and Chinese. Most of the subjects were married, attained at least a primary level of education, living with others and were not working. Notably, most of the respondents were from the lower strata of the income classification of poverty (≤RM970) based on the mean monthly income of these subjects, which was at RM959.96 ± 817.10 (approximately USD231.65).

The baseline characteristics of the subjects according to their frailty status are presented in Table 1. The prevalence of frailty was 15.9%, with a majority of the respondents found to be in the prefrail category (72.8%). Only 11.3% of them were healthy. Although there was no significant difference between the genders, a higher proportion of females were reported to be frail (17.7%) compared to the males (11.9%). The frailty status worsened with the increase in age (*p* < 0.001). However, there was no significant difference in frailty status observed across the ethnicities. The mean monthly income among subjects who were frail was found to be significantly lower when compared to their counterparts (*p* < 0.001), suggesting that frail syndrome was greater with reduced financial income. Subjects with frailty status were found to have at least one chronic disease (*p* < 0.05), greater BMI and waist circumference. In addition, a higher percentage of body fat, visceral fat, lower skeletal muscle mass and index (*p* < 0.001) and at risk of malnutrition (*p* < 0.01) were also prevalent among frail individuals. 

Further observation of the MNA indicators with the frailty status revealed that signs of reduced food intake (*p* < 0.05), presence of weight loss (*p* < 0.05) and immobility (*p* < 0.01) were significantly dominant among frail individuals. No significant differences were observed for psychological stress and neuropsychological problems for subjects with frailty status. 

### 3.2. Baseline Characteristic of the Subjects and Malnutrition Status

The malnutrition status of the subjects was measured using MNA-SF and is presented in Table 2. The classification of the malnutrition status was then segregated into well-nourished and at risk of malnutrition for easier interpretation. Most of the subjects were well-nourished, with 29.6% at risk of malnutrition and 3.3% malnourished. Based on the results on gender, about 33.5% of females and 31.5% of males were at risk of malnutrition. Those who were identified in this category were diagnosed with at least a chronic disease, weighed higher and had lower BMI compared to counterparts (*p* < 0.05). No significant difference was found between malnutrition across gender and ethnicity. However, those who were at risk of malnutrition earned less monthly income, though the result was proven to be insignificant (*p* > 0.05). Based on results for circumferences of body parts, both MUAC and CC were found to be significantly low compared to well-nourished individuals (*p* < 0.05). Skeletal muscle mass and index both showed a significantly lower mean among subjects identified as at risk of malnutrition. 

Further exploration on the phenotypes of frailty with malnutrition risk showed that there were significant differences observed for shrinking and exhaustion phenotypes with malnutrition status (*p* < 0.05). The prevalence of shrinking and exhaustion was significantly higher among subjects who were at risk of malnutrition. Nonetheless, there were no significant differences found in phenotypes on weakness, slowness and physical inactivity with malnutrition.

### 3.3. Association of Covariates with Frailty and Malnutrition 

Results from the multivariate analysis in relation to frailty status (Table 3) showed that increased age (odds ration (OR) = 1.192, CI = 1.063–1.338), body fat (OR = 0.904, CI = 0.826–0.991), skeletal muscle mass (OR = 0.683, CI = 0.535–0.871), skeletal muscle index (OR = 2.986, CI = 1.650–5.405) and malnutrition (OR = 1.589, CI = 0.988–4.161) were significant predictors of prefrailty. On the other hand, age (OR = 1.309, CI = 1.153–1.487), body fat (OR = 0.857, CI = 0.760–0.967), skeletal muscle index (OR = 5.098, CI = 1.624–16.009) and malnutrition were significant predictors of frailty. 

Results from the multivariate analysis in relation to malnutrition risk (Table 4) showed that lower MUAC (OR = 0.886, CI = 0.808–0.972), skeletal muscle (OR = 1.946, CI = 1.775–2.133), and frail were significant predictors of malnutrition. The present study revealed that frailty is the strongest predictor of malnutrition (OR = 5.273, *p* < 0.01), for which those who were frail were five times more likely, and those who were prefrail were twice as likely to be at risk of malnutrition. 

## 4. Discussion

Despite the extensive literature in this field of research, this study is currently the only study that explores the relationship between malnutrition and frailty among community-dwelling older adults in Malaysia. Therefore, this study provides new insights into the nutritional status of the frail community-dwellers in Malaysia. Results show that those who are physically frail are found to be of higher mean age, have lower household income and SMI and are at risk of malnutrition (*p* < 0.05). Similarly, those who are at risk of malnutrition have a higher number of chronic diseases, lower body weight and BMI, a lower circumference of arm and calf as well as lower skeletal muscle mass and frailty (*p* < 0.05). 

Hence, this present study demonstrates that there is a strong association between malnutrition and frailty. Among those who are frail, almost half of them are found to be at risk of malnutrition. The proportion of individuals suffering from poor nutritional status increased gradually with the growing level of frailty (*p* < 0.01), which has been deliberately reported previously [3,9,26]. Within the community setting, almost half of the frail elders are at a high risk of malnutrition. Moreover, 90% of the older adults with malnutrition are at high risk of frailty [4]. This relationship is further substantiated using the meta-analysis, whereby two-thirds of the malnourished individuals are found to be physically frail and one-quarter of them were prefrail [27]. It has been reported that malnourished and those at risk of malnutrition are four times more likely to be at high risk of frailty malnutrition, often prominent among those aged 65 years and above [3]. Several studies have identified substantial evidence of a positive association between malnutrition and physical functions [9], which strongly suggest both entities to be interrelated but of a distinct construct.

In this study, further explorations of the components in the criteria of frailty and MNA have revealed that both exhibit considerable overlapped constituents. We showed that there was a strong relationship between each of the MNA-SF items and frailty status. Both frailty and malnutrition are significantly related to each other, particularly for the phenotype of shrinking and exhaustion in the frailty criteria with reduced food intake, presence of weight loss and immobility in the components of malnutrition (*p* < 0.05). Thus, the phenotype of shrinking is found to reflect similar constructs in the component on the presence of weight loss in MNA, suggesting a strong correlation between these two components. This finding reiterates the previous reports that the most apparent overlap is the mutual assessment of weight loss [4,24]. The study which has employed the full length of MNA has also found a significant association between 12 out of 18 items in MNA with frailty [4], with the most apparent correspondence in the MNA constructs to be anorexia, weight loss, impaired mobility and psychological problem.

On the other hand, the phenotype of slowness is partly emulating the component of immobility in MNA. The assessment of immobility is dependent on the gait speed, while in the MNA component, it is dependent on self-report. The frailty criterion of “exhaustion” is evaluated by self-perception of “everything was an effort” or “I could not get going”, which inevitably shows some overlap with the depressive state in the neuropsychological item of MNA [4]. Though distinct, both assessments are deliberating upon similar factors. Frail elderly experienced muscle protein catabolism which impaired mobility and increased dependency on others. With the addendum consequences of malnutrition, both precipitate the onset of weight loss, reduced muscle quality, greater loss of appetite and therefore reduced the energy reserved for activities daily living, which leads to exhaustion and slowness. 

The pathophysiology of malnutrition and physical frailty is known to share common pathways and has been described as un-interchangeable [12]. Nonetheless, it is still a challenge to fully gauge the degree of observable association between nutritional and frailty state transitions due to the various measure of these two constructs. The imbricate pathophysiological pathway is apparent with similar phenotypes of weight loss and shrinking. The study has concluded that such cooccurrence may be attributed to the decrease of body tissues, followed by wasting, which is the most frequent phenotype of geriatric outcomes, including frailty and malnutrition [12]. In other studies, these conditions have been postulated to potentially exacerbate each other, which can lead to worsening conditions [28]. This similarity, however, is not only limited to the component of weight loss as both constructs share common sociodemography, physical and cognitive risk factors [3]. As proposed by Fried, the senescence musculoskeletal degeneration reduces the isometric strength of the hand or speed in walking, leading to less engagement in the functional activity and total energy expenditure. Reduced energy use will cause neuroendocrine dysregulation, resulting in anorexia of ageing that affects the nutritional status of older adults [1]. 

From another perspective, the roles of muscle mass and sarcopenia are vital in understanding malnutrition and frailty better. In this study, there is a significant relationship between the muscle mass with frailty and malnutrition, with reduced skeletal muscle mass when both constructs worsen. The finding from this study is supportive of the proposed vicious cycle of frailty, which highlighted sarcopenia as one of the main contributors of frailty [29]. As an individual ages, skeletal muscle undergoes quality and quantity alteration, which leads to a progressive loss of muscle mass and strength [11] and is often treated as the primary component of sarcopenia. The clinical hallmark characteristic of frailty involves a poor functional capacity, not all of which are related to skeletal muscle amount or function [16], as in the sarcopenia construct. The accumulation of deficits in wider perspective is the integral component that differentiates frailty from sarcopenia. As discussed in a previous study [23], malnutrition has been identified as the key predictors of sarcopenia. The finding is inarguable since both frailty and sarcopenia show a significant association and overlap with each other as muscle strength is part of the physical frailty components. 

Studies have included the assessment of muscle function and quality as a linking substrate between malnutrition and physical ability [30,31]. Reduced muscle strength and increased skeletal muscle loss precipitate the onset of frailty and malnutrition. The few studies which have addressed this relationship corroborated similar evidence as well [10,25]. Besides, recent iterations have proposed that reduced muscle strength measured by handgrip strength is an appropriate identification measure of frailty [12,32,33]. However, the prognostic marker of handgrip strength is not only limited to frailty; but, also often used as a proxy of nutritional status, which demonstrates decreased muscle function with nutritional deprivation [34]. Multiple lines of evidence from studies have also shown that a decrease in muscle power is not only associated with poor prognosis in malnutrition but is also a direct predictor of poor physical performance [35]. Thus, the inclusion of muscle-mass assessment is by far crucial to capture the cooccurrence and distinct characteristics of frailty and malnutrition among the older population. 

This study has identified some limitations. Firstly, the sample population is urban-living older adults, which may not represent the general population when extrapolated. This cross-sectional study design is also unable to signify the cause and effects of the tested variables. Nevertheless, this study is able to determine the frailty incidence and establish the association of malnutrition among the elderly population in a primary health care setting.

## 5. Conclusions

In conclusion, the use of MNA is practical in the identification of frailty syndrome among older adults in a community care setting. As such, the adaptation of MNA as an indirect tool to screen for frailty syndrome is promising. Given that the prevalence of malnutrition is much higher than frailty, this result strongly supports that malnutrition and physical frailty are interrelated, but possess distinct syndromes. The present study has addressed the relationship between malnutrition and frailty among community-dwelling older adults using past studies within this area of research. Since frailty in older adults is of rising concern, there is a need to prevent and manage frailty by concentrating on the essential factors, such as maintaining good nutritional status and physical function to decrease the escalation of frailty syndrome. The implementation is especially important for primary health care to be amenable in governing this epidemic at an earlier stage. More insights are warranted to account for potential biases that may have been present in this study. 

## Figures and Tables

**Table 1:** Baseline characteristic of older adults stratified by frailty status, *n* (%).

Characteristics			*n* = 301		
	Total (*n* = 301)	Normal (*n* = 34)	Prefrail (*n* = 219)	Frail (*n* = 48)	*p*-Value
Age (years) (mean ± SD)		63.94 ± 3.25	67.05 ± 5.32	69.44 ± 6.61	0.000 ***^a^
Sex					0.117 ^b^
Male	92 (30.6)	15 (44.1)	66 (30.1)	11 (22.9)	
Female	209 (69.4)	19 (55.9)	153 (69.9)	37 (77.1)	
Ethnicity					0.224 ^b^
Malay	213 (70.8)	19 (55.9)	159 (72.6)	35 (72.9)	
Chinese	37 (12.3)	8 (23.5)	23 (10.5)	6 (12.5)	
Indian	45 (15.0)	7 (20.6)	37 (16.9)	7 (14.6)	
Monthly income (RM), median (IQR)	800.0 (700.0)	1000.0 (1125.0)	700.0 (764.0)	500.0 (687.5)	0.000 ***^c^
Chronic disease					0.023 *^b^
None	89 (29.6)	14 (41.2)	68 (31.1)	7 (14.6)	
≥1 chronic disease	212 (70.4)	20 (58.8)	151 (68.9)	41 (85.4)	
Anthropometric measurement					
Weight (kg)	65.5 ± 13.9	68.45 ± 3.77	64.82 ± 12.32	66.31 ± 19.5	0.328 ^a^
Body Mass Index (kgm^−2^)	27.5 ± 5.5	26.74 ± 4.47	27.53 ± 4.84	28.03 ± 27.52	0.567 ^a^
Body Part Circumference					
WC (cm)	92.1 ± 13.3	89.81 ± 11.96	91.85 ± 11.75	95.023 ± 19.57	0.184 ^a^
MUAC (cm)	29.7 ± 4.2	29.29 ± 3.87	29.69 ± 3.80	29.98 ± 5.97	0.769 ^a^
CC (cm)	34.3 ± 4.6	34.19 ± 5.61	34.32 ± 4.19	34.22 ± 5.53	0.981 ^a^
Body Composition					
Body Fat percentage (%)	36.1 ± 6.5	33.73 ± 6.61	36.31 ± 6.30	36.98 ± 7.06	0.060 ^a^
Visceral Fat, median (IQR)	12.5 (8.3)	11.0 (7.0)	12.5 (8.5)	13.0 (10.1)	0.639 ^c^
Skeletal muscle mass (kg)	28.1 ± 7.8	30.71 ± 8.88	28.07 ± 7.64	24.69 ± 5.37	0.001 **^a^
Skeletal muscle index (kgm^−2^)	12.0 ± 3.8	13.14 ± 4.12	12.12 ± 3.82	9.73 ± 2.31	0.000 ***^a^
Malnutrition status					0.004 **^b^
Normal	202 (67.1)	27 (79.4)	152 (69.4)	23 (47.9)	
At risk of malnutrition	99 (32.9)	7 (20.6)	67 (30.6)	25 (52.1)	
Malnutrition Indicator					
Reduced in food intake	92 (30.6)	8 (23.5)	60 (27.4)	24 (50.0)	0.022 *^b^
Present of weight loss	209 (39.4)	11 (32.4)	85 (26.4)	23 (47.9)	0.026 *^b^
Immobility	10 (3.3)	1 (2.9)	4 (1.8)	5 (10.4)	0.002 **^b^
Psychological stress	54 (17.9)	6 (17.6)	35 (16.0)	13 (27.1)	0.192 ^b^
Neuropsychological problems	79 (26.2)	10 (29.4)	55 (25.1)	14 (29.2)	0.804 ^b^

^a^ Pearson product moment correlation; ^b^ chi-square test of association; ^c^ Spearman correlation statistical significance at 0.05 level (two-tailed). * Significant at level 0.05; ** Significant at level 0.01; *** Significant at level 0.001. SD = standard deviation; IQR = interquartile range.

**Table 2:** Baseline characteristic of older adults stratified by nutritional status.

Characteristics	*n* = 301		
	Well-Nourished (*n* = 202)	At Risk of Malnutrition (*n* = 99)	*p*-Value
Age (years)	66.66 ± 5.32	67.94 ± 5.88	0.060 ^a^
Sex			0.737 ^b^
Male	63 (31.2)	29 (29.3)	
Female	139 (68.8)	70 (70.7)	
Ethnicity			0.391 ^b^
Malay	148 (73.3)	65 (65.7)	
Chinese	23 (11.4)	14 (14.1)	
Indian	31 (15.3)	20 (20.2)	
Monthly income (RM), median (IQR)	800.0 (625.0)	600.0 (750.0)	0.185 ^c^
Chronic disease			0.002 **^b^
None	71 (35.1)	18 (18.2)	
≥1 chronic disease	131 (64.9)	81 (81.8)	
Anthropometric measurement			
Weight (kg)	67.29 ± 13.32	61.73 ± 14.27	0.001 **^a^
Body Mass Index (kgm^−2^)	28.21 ± 5.21	26.11 ± 5.78	0.002 **^a^
Body Part Circumference			
WC (cm)	93.15 ± 13.09	90.02 ± 13.68	0.055 ^a^
MUAC (cm)	30.24 ± 3.94	28.56 ± 4.56	0.001 **^a^
CC (cm)	34.73 ± 4.61	33.39 ± 4.40	0.016 *^a^
Body Composition			
Body Fat percentage (%)	36.34 ± 6.32	35.59 ± 6.85	0.314 ^a^
Visceral Fat, median (IQR)	14.0 (7.6)	10.5 (8.0)	0.073 ^c^
Skeletal muscle mass (kg)	28.87 ± 7.64	26.57 ± 7.81	0.016 *^a^
Skeletal muscle index (kgm^−2^)	12.31 ± 3.86	11.39 ± 3.69	0.049 *^a^
Frailty Status			0.004 **^b^
Robust	27 (13.4)	7 (7.1)	
Prefrail	152 (75.2)	67 (67.7)	
Frail	23 (11.4)	25 (25.3)	
Frailty Phenotype			
Shrinking	13 (6.4)	24 (24.2)	0.000 ***^b^
Exhaustion	34 (16.8)	27 (27.3)	0.034 * ^b^
Weakness	159 (78.7)	80 (80.8)	0.673 ^b^
Slowness	48 (23.8)	33 (33.3)	0.079 ^b^
Physical inactivity	37 (18.3)	21 (21.2)	0.550 ^b^

^a^ Pearson product moment correlation; ^b^ chi-square test of association; ^c^ Spearman correlation. Statistical significance at 0.05 level (two-tailed). * Significant at level 0.05; ** Significant at level 0.01; *** Significant at level 0.001. SD = standard deviation; IQR = interquartile range.

**Table 3:** Risk factors related to frailty status.

Predictor	Prefrail			Frail		
	OR	95% CI	*p*-Value	OR	95% CI	*p*-Value
Age	1.192	1.063–1.338	0.003	1.309	1.153–1.487	0.000
Monthly income (RM)	1.000	0.999–1.000	0.641	0.999	0.999–1.000	0.999
WC	1.021	0.980–1.063	0.330	1.042	0.986–1.101	0.143
MUAC	0.129	0.969–1.278	0.129	1.099	0.923–1.308	0.289
Body fat	0.904	0.826–0.991	0.031	0.857	0.760–0.967	0.012
Skeletal muscle mass	0.683	0.535–0.871	0.002	0.801	0.606–1.061	0.122
Skeletal muscle index	2.986	1.650–5.405	0.000	2.564	1.325–4.959	0.005
Malnutrition risk (0: at risk of malnutrition 1: well-nourished)	1.589	0.988–4.161	0.034	5.098	1.624–16.009	0.005

Reference category: normal. OR: odds ratio; CI: confidence interval. The reference category is 1.00. Statistical significance at 0.05 level (two-tailed).

**Table 4:** Risk factors related to malnutrition risk.

Predictor	At Risk of Malnutrition		
	OR	95% CI	*p*-Value
Age	1.00	0.950–1.052	0.995
Monthly income (RM)	0.974	0.670–1.415	0.889
WC	1.007	0.978–1.036	0.655
MUAC	0.886	0.808–0.972	0.010
Body fat	1.033	0.978–1.091	0.241
Skeletal muscle mass	1.946	1.775–2.133	0.000
Frailty status (0: Frail)	5.273	1.587–16.484	0.004
(1: Prefrail)	2.123	0.834–5.404	0.114

Reference category: normal. OR: odds ratio; CI: confidence interval. The reference category is 1.00. Statistical significance at 0.05 level (two-tailed).
